# Host Cells of *Leucocytozoon* (Haemosporida, Leucocytozoidae) Gametocytes, with Remarks on the Phylogenetic Importance of This Character

**DOI:** 10.3390/pathogens12050712

**Published:** 2023-05-13

**Authors:** Carolina Romeiro Fernandes Chagas, Mélanie Duc, Germán Alfredo Gutiérrez-Liberato, Gediminas Valkiūnas

**Affiliations:** Nature Research Centre, 01109 Vilnius, Lithuania; melanie.duc@gamtc.lt (M.D.); german.liberato@gamtc.lt (G.A.G.-L.); gediminas.valkiunas@gamtc.lt (G.V.)

**Keywords:** *cytb* lineage, erythrocytes, thrombocytes, lymphocytes, red blood cells, morphological features, *Parus*, *Phylloscopus*, *Sylvia*, *Turdus*

## Abstract

*Leucocytozoon* parasites remain poorly investigated in comparison to other haemosporidians. The host cell inhabited by their blood stages (gametocytes) remains insufficiently known. This study aimed to determine the blood cells inhabited by *Leucocytozoon* gametocytes in different species of Passeriformes and to test if this feature has a phylogenetic importance. We microscopically analyzed blood films stained with Giemsa from six different bird species and individuals and used PCR-based methods for parasite lineage identification. The DNA sequences obtained were applied for phylogenetic analysis. *Leucocytozoon* parasite from the song thrush *Turdus philomelos* (cytochrome *b* lineage STUR1), the blackbird *Turdus merula* (undetermined lineage), the garden warbler *Sylvia borin* (unknown lineage) inhabited erythrocytes, a parasite from the blue tit *Cyanistes caeruleus* (PARUS4) infects lymphocytes, while in the wood warbler *Phylloscopus sibilatrix* (WW6) and the common chiffchaff *Phylloscopus collybita* (AFR205) they were found inhabiting thrombocytes. Parasites infecting thrombocytes were closely related, while the parasites infecting erythrocytes were placed in three different clades, and the one found in lymphocytes was placed in a separate clade. This shows that the determination of host cells inhabited by *Leucocytozoon* parasites can be phylogenetically important and should be considered in future species descriptions. Noteworthy, phylogenetic analysis might be used for the prediction of which host cells parasite lineages might inhabit.

## 1. Introduction

*Leucocytozoon* (Leucocytozoidae, Haemosporida) is an insufficiently investigated group of avian haemosporidian parasites. Even though it contains approximately 50 morphologically described species [1,2], these parasites’ genetic diversity is considerably high, with 1429 cytochrome *b* gene (*cytb*) lineages reported; however, only 25 of them were linked to 15 morphospecies (MalAvi database, http://130.235.244.92/Malavi/ accessed on 1 October 2022) [3].

Compared to the other haemosporidian parasites (*Plasmodium* and *Haemoproteus*)*, Leucocytozoon* species are still insufficiently investigated, and little progress has been made in the last decade regarding species description and linking *cytb* lineages to morphospecies [1,4]. This is mainly because the morphological characterization of *Leucocytozoon* parasites is challenging due to the presence of very few features in the developing and mature gametocytes that could be used for species identification [4,5]. Additionally, even if some parasite species can be readily identified, it is a common case that these features do not show enough divergence to describe a new parasite species in similar host-parasite associations on cellular levels. It is also necessary to mention that these parasites are frequently found in low parasitemia and deformed in the blood films, which interfere directly with the morphological analysis, parasite species description, and identification [5].

It is likely that many more *Leucocytozoon* species exist, but they cannot be identified based solely on the morphological characteristics of blood stages, requiring studies about other aspects of their life cycle [4]. Even though studies addressing different aspects of *Leucocytozoon* parasites’ life cycle, such as the identification of vectors [6,7,8] and the exoerythrocytic development [9,10,11,12,13], have been conducted, it has been happening at a slower pace when compared with the closely related haemosporidian, *Plasmodium* and *Haemoproteus* [13,14].

There is also another important issue that needs to be addressed for *Leucocytozoon*, due to the possible relationship to their evolution and taxonomy. Mainly, which host blood cells do these parasites inhabit? For *Plasmodium* and *Haemoproteus*, this topic is solved, mainly because their blood stages often do not deform the infected host cell from the early stages of development, and the host cell can be readily identified [5]. However, *Leucocytozoon* parasites have a big influence on infected host cells, deforming them even at the early stages of development [5]. It is known that some *Leucocytozoon* species can develop in erythrocytes [5], while others were reported to inhabit thrombocytes [2,15] and even mononuclear leukocytes [5,16]. However, we still do not know if the host cells inhabited by the parasites are specific to parasite species, or host species, or if there are some other factors, which are involved in the selection to inhabit certain blood cells. This study aimed to investigate the type of blood cells inhabited by different *Leucocytozoon* parasites in different naturally infected species of Passeriformes and to test if this feature has a phylogenetic importance.

## 2. Materials and Methods

### 2.1. Sample Selection and Microscopical Analysis

The *Leucocytozoon* spp. infected samples were selected from the available collection of the Nature Research Centre based on the presence of intracellular merozoites or young gametocytes in the Giemsa-stained blood films. These samples were all collected in Ventės Ragas Ornithological Station (55°20′38.93″ N, 21°11′34.05″ E), in Lithuania, during Spring (May 2016 and 2019) and Autumn (September 2020) bird migrations. Briefly, the birds were caught using funnel traps, mist nets, and ‘zigzag’ traps. The blood was withdrawn by puncturing the brachial vein with a needle and collected using heparinized capillary tubes. One small drop of blood was used to prepare blood films, which were fixed in absolute methanol for 1–2 s and stained with a 10% Giemsa solution for 1 h [17]. The remaining blood was transferred to a tube with SET buffer (0.05 M Tris, 0.15 M NaCl, 0.5 M EDTA, pH 8.0), which was used to confirm parasite lineage by molecular techniques.

All microscopic analyses were conducted using a light microscope Olympus BX41TF equipped with an Olympus DP12 digital camera and the image software Olympus DP-SOFT (Olympus, Tokyo, Japan). The blood films were examined for 15–20 min at low magnification (×400), and then at least 100 fields were studied at high magnification (×1000) for the presence of *Leucocytozoon* parasites. To confirm which host blood cells (erythrocytes, thrombocytes, or different leukocytes) were infected, the images of intracellular merozoites and young and mature gametocytes were collected. In parallel, the images of all different types of non-infected blood cells were collected for comparison purposes (Supplementary Figure S1). Identification of host cells was carried out according to Clark et al. [18]. Voucher preparations of the used material were deposited in the Nature Research Centre, Vilnius, Lithuania (accessions 49373NS, 49374NS, 49481NS-49490NS).

### 2.2. DNA Extraction, PCR, Sequencing, and Parasites Lineage Identification

DNA of the selected samples was extracted using an ammonium acetate protocol. Briefly, proteinase K (Thermo Scientific™, Vilnius, Lithuania) and SDS 20% were added to the blood samples, which were incubated at 56 °C overnight. Then, ammonium acetate was added to the tubes and incubated in a shaker at 800 rpm at room temperature for 1 h. After that, samples were centrifuged at 10,000 rpm for 10 min. The supernatant was transferred to a new tube and ethanol 96% was added to it. Then, samples were centrifuged again at 10,000 rpm for 15 min. Finally, the supernatant ethanol was removed, and samples were rinsed with 70% ethanol. Obtained DNA was dissolved in TE buffer [19].

A nested PCR protocol was used to amplify a ~480 bp fragment of *cytb* of haemosporidian parasites. In the first reaction, primers HaemNFI/HaemNR3 amplified the DNA of *Plasmodium*, *Haemoproteus*, and *Leucocytozoon*. In the second reaction, primer sets HaemF/HaemR2 and HaemFL/HaemR2L were used to amplify *Plasmodium*/*Haemoproteus* and *Leucocytozoon*, respectively [20]. One positive (sample with *Leucocytozoon* infection confirmed by microscopy) and one negative (ultrapure water) control were used in every PCR reaction. Amplification was confirmed by electrophoresis in a 2% agarose gel. Positive samples were sequenced from both ends with the respective primers, using Big Dye Terminator V3.1 Cycle Sequencing Kit and ABI PRISM^TM^ 3100 capillary sequencing robot (Applied Biosystems, Foster City, CA, USA).

Obtained sequences were evaluated and aligned to generate a consensus sequence using Geneious Prime 2022.0.1 software (https://www.geneious.com, accessed on 1 December 2022). Samples positive for more than one parasite genus and with double peaks in the electropherograms were considered co-infections. Consensus sequences were compared to other sequences deposited in the MalAvi database using the BLAST tool (http://130.235.244.92/Malavi/, accessed on 1 March 2023 [3]). If the amplified lineage had a 100% match with available sequences, the parasite was considered to belong to this lineage; new lineages were identified when one bp of difference was found between our sequences and the deposited ones. All obtained parasite sequences were deposited in GenBank (OK646333, OK646334, OQ784302-OQ784305) and MalAvi database.

### 2.3. Phylogenetic Analysis

A Bayesian inference (BI) phylogeny was carried out using MrBayes version 3.1.2 [21], implemented in the platform CIPRES Science Gateway V3.3 [22]. An alignment was constructed based on 104 sequences with 479 bp length. DNA sequences of other haemosporidians (*Plasmodium*, *Leucocytozoon* (*Akiba*), and *Haemoproteus*) were used as an outgroup. The alignment was constructed in MEGA 11 V11.0.13 [23] and aligned with MAFFT V7 [24]. The analysis was performed under the general time-reversal model (GTR + I + G) suggested by jModelTest 2.1.1 [25] as the best of 88 models according to the corrected information criterion of Akaike (AICc). For BI independent Markov Chain Monte Carlo (MCMC) simulations were run simultaneously with six chains using 1 × 10^7^ generations sampled every 500 generations. After discarding 25% of the trees as “burn-in”, the remaining trees were used to build the majority rule consensus tree, which was visualized and edited using FigTree version 1.4.3 [26] and MEGA 11 V11.0.13 [23].

## 3. Results

In total, 50 samples from different individuals were analyzed. Samples from six bird individuals and species were selected: a song thrush *Turdus philomelos*, a blackbird *Turdus merula*, a garden warbler *Sylvia borin*, a blue tit *Cyanistes caeruleus*, a wood warbler *Phylloscopus sibilatrix*, and a common chiffchaff *Phylloscopus collybita*. The parasite lineages were determined in all these birds, except for the garden warbler due to the absence of a blood sample to be used in the molecular analysis. All *Leucocytozoon* lineages recovered have been reported before and have not been linked to morphospecies (Table 1).

**Figure 1:**
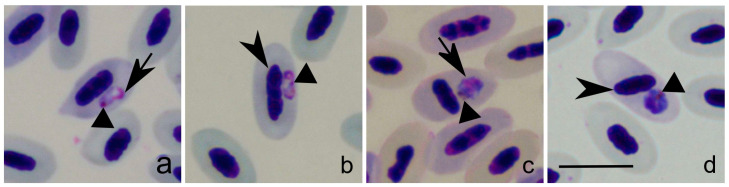
Young intraerythrocytic stages of *H. minutus* (**a**,**b**) and *P. matutinum* (**c**,**d**) from blackbird *T. merula*. Simple short arrows, parasite nuclei. Simple arrowheads, host cell nuclei. Triangle arrowheads, pigment granules. Methanol-fixed and Giemsa-stained. Scale bar = 10 μm.

**Figure 2:**
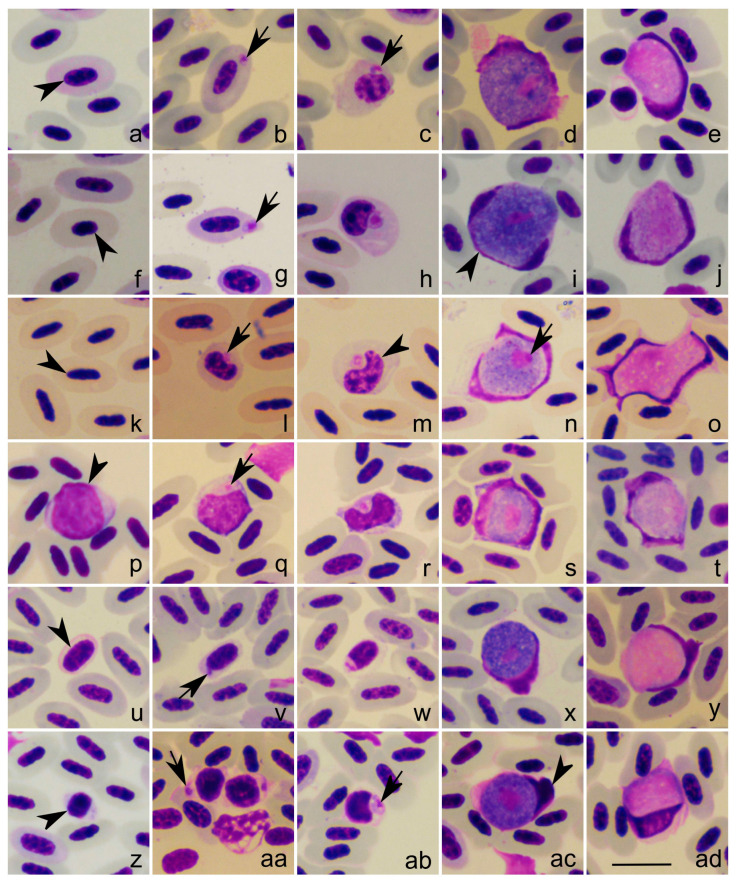
*Leucocytozoon* parasites infecting a song thrush *T. philomelos* (cytochrome *b* lineage STUR1) (**a**–**e**), a blackbird *T. merula* (unknown lineage) (**f**–**j**), a garden warbler *S. borin* (unknown lineage) (**k**–**o**), a blue tit *C. caeruleus* (PARUS4) (**p**–**t**), a wood warbler *P. sibilatrix* (WW6) (**u**–**y**), and a common chiffchaff *P. collybita* (AFR205) (**z**–**ad**). Non-infected erythrocytes (**a**,**f**,**k**), lymphocyte (**p**), and thrombocytes (**u**,**z**). Merozoites (**b**,**g**,**l**,**q**,**v**,**aa**) and growing gametocytes (**c**,**h**,**m**,**r**,**w**,**ab**). Mature macrogametocytes (**d**,**i**,**n**,**s**,**x**,**ac**) and microgametocytes (**e**,**j**,**o**,**t**,**y**,**ad**). Simple short arrows, parasite nuclei. Simple arrowheads, host cell nuclei. Methanol-fixed and Giemsa-stained blood films. Scale bar = 10 μm.

Co-infections with other haemosporidian parasites were detected in all studied birds, including different lineages belonging to *Leucocytozoon* (Table 1). Young stages of *Leucocytozoon* parasites can be readily morphologically distinguished from young stages of *Plasmodium* and *Haemoproteus* mainly due to the lack of pigment granules (compare Figure 1a–d with Figure 2b,g,l,q,v,aa), which are visible even in smallest gametocytes and trophozoites of these parasites (Figure 1a). However, some other differences were also seen in the parasite lineages used in this study, even though they might not be so evident in all *Plasmodium* and/or *Haemoproteus* species, such as (i) the smooth edges in *Leucocytozoon* parasite early stages (compare Figure 1c with Figure 2b,g); (ii) the predominantly roundish *Leucocytozoon* parasite form in infected host blood cells (compare Figure 1 with Figure 2b,g,l,q,v,aa); (iii) the centrally located *Leucocytozoon* parasite nucleus when infecting erythrocytes (compare Figure 1a–d with Figure 2b,g,l) [5].

*Leucocytozoon* parasites were seen infecting three different types of blood cells: erythrocytes (Figure 2a–o), lymphocytes (Figure 2p–t), and thrombocytes (Figure 2u–ad) (Table 1). Mature erythrocytes can be identified by the presence of an evenly colored eosinophilic cytoplasm, and an ovoid nucleus with condensed chromatin (Figure 2f,k); while polychromatic erythrocytes are characterized by a cytoplasm with a bluish coloration and less condensed chromatin than mature erythrocytes (Figure 2a) [18]. Moreover, lymphocytes can be identified based on the presence of a roundish nucleus, more or less condensed chromatin, and a small rim of basophilic cytoplasm (Figure 2p) [18]. Thrombocytes can be identified by the presence of a very dense, darkly stained nucleus with small to moderate colorless cytoplasm (Figure 2u,v) [18]. These characteristics were maintained in cells infected by young stages of *Leucocytozoon* (compare Figure 2 and Supplementary Figure S1).

Merozoites and growing gametocytes of *Leucocytozoon* parasites that develop in erythrocytes usually have a roundish or oval form, with a big roundish nucleus (Figure 2b,c,g,h,l,m). They were found in polychromatic erythrocytes in the song thrush (parasite *cytb* lineage STUR1) (Figure 2a–e); in mature erythrocytes in the blackbird (undetermined lineage) (Figure 2f–j) and in the garden warbler (unknown lineage) (Figure 2k–o). The parasites from the blue tit (PARUS4), that develops in thrombocytes, also presented similar features as the ones developing in erythrocytes (Figure 2p–t). The *Leucocytozoon* parasites infecting thrombocytes, merozoites and young gametocytes are also roundish; however, they have a smaller size in comparison to the ones developing in erythrocytes, and smaller nuclei with a roundish to elongated form (Figure 1v,w,aa,ab). They were present in the wood warbler (WW6) (Figure 2u–y) and in the common chiffchaff (AFR205) (Figure 2z–ad). Host cell identification was possible due to the comparison between cells infected with the earliest *Leucocytozoon* stages (merozoites and early gametocytes) (Figure 2b,g,l,q,v,aa) and non-infected cells (Figure 2a,f,k,p,u,z and Supplementary Figure S1).

The morphological identification of *Leucocytozoon* parasites was difficult in all studied birds, and the parasite species could not be confirmed or linked to any parasite lineage recovered. This was due to several factors: (i) the *Leucocytozoon* co-infection; (ii) the low parasitemia; and (iii) the predominant presence of deformed gametocytes in the blood films (Figure 2o,t), which is a frequent case in *Leucocytozoon* infections. All parasites found in studied birds deform the host cell into a roundish host-parasite complex (Figure 2).

Even though we could not identify species of found parasites, they all showed features that correspond to already described morphospecies. In the song thrush, the blackbird, and the garden warbler observed mature gametocytes are similar to *Leucocytozoon dubreuili*. Parasite nuclei were more or less dumbbell-shaped with thickening at both ends and with the nuclei extending more than half of the circumference of gametocytes (Figure 2d,e,i,j,n,o) [5]. While the *Leucocytozoon* parasite infecting the blue tit had morphological features compatible with *Leucocytozoon majoris*, whose nuclei of the host cell are of band-like form and have approximately the same width along all its length; the nucleus extends more than half of the circumference of the gametocyte (Figure 2s,t) [5]. For the parasites seen in the wood warbler and the common chiffchaff, mature gametocytes have morphological features similar to *Leucocytozoon fringillinarum*. Mainly, the nucleus of the host cell assumes a cap-like form, sometimes band-like in shape; the nucleus usually extends less than half of the circumference of the gametocyte (Figure 2x,y,ac,ad) [5].

**Figure 3:**
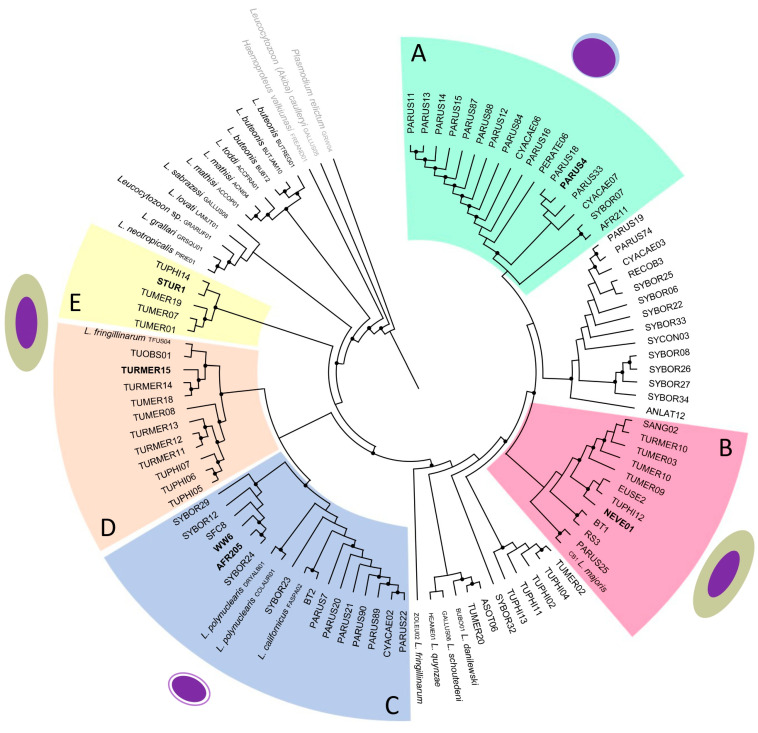
Bayesian inference phylogeny of *Leucocytozoon* parasites found in the present study (in bold). The outgroup is indicated in light grey letters. Clade **A** indicates parasite lineages that infect Paridae birds and that we found infecting lymphocytes (there is no information about host cells of other parasite lineages present in this clade). Clade **B** indicates parasite lineages that infect mainly Turdidae birds. Clade **C** highlights the clade where parasites were found infecting thrombocytes (WW6, AFR205, and *Leucocytozoon polynuclearis*, there is no information about the other parasite lineages and species present in this clade). Clades **D** and **E** indicate parasite lineages that infect Turdidae birds. Nodes with a posterior probability of ≥80% are indicated with dots. GenBank accession numbers are given in Supplementary Table S1, and parasite lineages are given according to the MalAvi database.

The phylogenetic analysis (Figure 3) showed that all parasite lineages and species reported to be inhabiting thrombocytes clustered together with *Leucocytozoon polynuclearis*, a *Leucocytozoon* parasite described in Piciformes and that infects such cells (Figure 3, clade C). Parasite lineage found infecting lymphocytes was placed in a separate clade (Figure 3, clade A). The other parasite lineages and species reported to inhabit erythrocytes are distributed randomly in the tree (Figure 3, clades B, D, and E). *Leucocytozoon* sp. STUR1, from the song thrush, clustered with other parasite lineages reported in Turdidae birds (Figure 3, clade E). Parasites lineages recovered from the blackbird were placed in two different clades, with TURMER15 being closely related to the lineage *L. fringillinarum* TFUS04 isolated from *Turdus fuscater* from Colombia (Figure 3, clade D) and NEVE01 closely related to other *Leucocytozoon* lineages from Turdidae birds isolated from European birds (Figure 3, clade B). Parasite lineage PARUS4, recovered from the blue tit was placed in a clade together with other *Leucocytozoon* lineages isolated from Paridae birds (Figure 3, clade A), but far from the *L. majoris* CB1 (Figure 3, clade B).

## 4. Discussion

The key result of this study is that *Leucocytozoon* gametocytes can develop in different types of host cells, such as erythrocytes, lymphocytes, and thrombocytes. Parasites morphologically similar to *L. dubreuili* were found infecting erythrocytes, the one similar to *L. majoris* was found infecting lymphocytes, while those similar to *L. fringillinarum* were present in thrombocytes. Phylogenetic analysis (Figure 3) showed that the parasite lineages, in which gametocytes deform the host cell into a roundish host-parasite complex and inhabit thrombocytes clustered together, and the one infecting lymphocytes was placed in a separate clade, this might be an indication that they probably represent an independent line of *Leucocytozoon* evolution.

Early stages of *Leucocytozoon* parasites could be readily distinguished from the closely related *Plasmodium* and *Haemoproteus* (compare Figure 1 with Figure 2). This is mainly due to the lack of pigment granules in *Leucocytozoon* parasites, a feature that is seen even in young stages of *Plasmodium* and *Haemoproteus* [5]. That said, co-infections do not represent a problem in the identification of merozoites and young gametocytes of *Leucocytozoon* by microscopy.

*Leucocytozoon* parasites were shown to infect various blood cells, including erythrocytes, thrombocytes, and mononuclear leukocytes [2,5,15,16,27]. Most of these reports were based on microscopic examination of blood films stained with Giemsa, and the origin of the infected cells was determined based on the comparison of morphological features of non-infected and infected blood cells [2,5,16]. There is only one study with chicken parasites that applied immunofluorescence techniques to identify host blood cells inhabited by *Leucocytozoon* sp. [15]. However, the application of such a technique is not so straightforward in wild birds and markedly differs in each avian host and their *Leucocytozoon* parasites. To begin with, the specificity of commercially available antibodies is well-known for poultry, but not for wild bird species, for which such antibodies are absent. The application of chicken antibodies can produce false negative or ambiguous results during wildlife studies. In addition, tests are necessary to validate the application of available antibodies for cell identification in wild birds. Furthermore, this is an expensive technique that requires specific reagents and equipment, which might not be easily available in laboratories dealing with wildlife diversity research. Thus, even though immunofluorescence techniques would be an ideal methodology for host cell identification, our experience shows that much development is needed before this methodology could be widely applied using wild bird samples (C.R.F.C., personal observation). The morphological identification of infected cells using microscopic examination remains useful at this stage of research. Groff et al. [2] used blood films stained with Giemsa to determine the origin of host cells inhabited by *L. polynuclearis*. The same protocol was used in the present study and produced reliable results.

Blood host cells inhabited by *Leucocytozoon* parasites were identified only for a few parasite species, with most studies not reporting this information in the parasite description or the host cell is just mentioned and images are not provided [5,28,29,30,31,32,33,34,35,36]. This is not surprising, since the merozoites and young gametocytes of *Leucocytozoon* are not commonly observed in blood films of naturally infected wild birds. When present, they deform the infected host cell significantly, making the identification of such host cells difficult. As a result, the description of new *Leucocytozoon* species is based on the morphological features of mature gametocytes [4,5], not considering the host cell infected by the parasite. Most described *Leucocytozoon* species seem to inhabit erythrocytes [5,16,35,37], fewer inhabit mononuclear leukocytes [5,16,28,28,34], and only two have been confirmed to inhabit thrombocytes [2,15]. The present study adds three lineages to the list of *Leucocytozoon* parasites that can develop in erythrocytes (STUR1, TURMER15, NEVE01), one lineage that can develop in lymphocytes (PARUS4), and two lineages that can develop in thrombocytes (WW6 and AFR205).

Despite the morphological resemblance between parasites found in the present study and morphologically described *Leucocytozoon* species, it was difficult to identify them using morphological characters of blood stages. Unfortunately, this is not an exception and many other studies present only molecular data about the *Leucocytozoon* parasites found in wild birds, without microscopical analysis [38,39,40,41,42,43]. It is undeniable that the use of molecular-based techniques improved the diagnosis of several parasites, making it reliable and allowing the assessment of genetic diversity and worldwide distribution [44]. However, these methods are not able to distinguish between different parasite life cycle stages and can only point out the presence of parasite DNA in the tested sample. A good example of this limitation is the application of PCR-based methods in investigations of haemosporidian vectors [14]. If the tested insect is PCR-positive, it does not confirm that the parasite can complete its development in it, which can be accessed only by confirming the presence of infective stages in their salivary glands’ preparations [14].

Morphological analysis for parasite diagnosis continues to be the gold standard method in many cases, is cost-effective, and might be the only accurate way to identify certain parasites and their stages in analyzed samples [44]. For example, it is well known that some molecular-based methods underestimate the prevalence of single and co-infections of certain haemosporidian parasites [45,46]. For *Leucocytozoon* parasites, recent studies have shown that morphological analysis underestimates species diversity, while *cytb* data likely overestimate species diversity [4,47]. That said, it is essential to combine morphological and molecular methods during protist parasite research [48].

*Leucocytozoon dubreuili* has not been genetically characterized yet, even though it is a parasite species that was described more than a century ago, probably in *Turdus* sp. wintering in Vietnam [5]. The original description does not specify the bird species, referencing it only as “grive”, which can be translated as “thrush”. However, there are more than 15 Turdidae species known in the region, making it difficult, if not impossible, to find the same host species and to molecularly characterize this *Leucocytozoon* species. Additionally, there are reports of *L. dubreuili* in more than 60 species of birds, likely representing cryptic species, that are morphologically similar to the parasites first described in Vietnam [5]. In our study, three birds had parasite lineages matching *L. dubreuili* morphotype, the song thrush, the blackbird, and the garden warbler (Figure 2a–o). *Leucocytozoon* lineage found in the song thrush (STUR1) clustered with other lineages described in blackbirds and song thrushes (Figure 3, clade E), but none of them have been linked to morphospecies. Furthermore, the blackbird had a co-infection with two different *Leucocytozoon* lineages (NEVE01 and TURMER15), and it was not possible to link the parasite in the blood smear with a certain *cytb* lineage. However, these two lineages were positioned in different clades in our phylogenetic analysis, with TURMER15 being closely related to *L. fringillinarum* TFUS04 (Figure 3, clade D); and NEVE01 being closely related to other lineages described in Turdidae birds from Austria and the Czech Republic (Figure 3, clade B) [4]. This highlights how challenging it can be to work with *Leucocytozoon* parasites and link older parasite descriptions, based on morphological features and host species, to their genetic lineage.

*Leucocytozoon* parasite found in the blue tit was morphologically similar to *L. majoris* (Figure 2p–t). In the phylogenetic analysis, this lineage (PARUS4) was placed together with other lineages described in *Parus* birds (Figure 3, clade A), but far from *L. majoris* CB1 (Figure 3, clade B). Even though *L. majoris* was morphologically described in a *Parus major* (Paridae) in France [5], this morphospecies was linked to a lineage from a sample collected in the United States, from a Mountain White-Crowned Sparrow (*Zonotrichia leucophrys oriantha*) [49]. This bird species belongs to Fringillidae and is from a different zoogeographical region than the hapantotype, and maybe this identification is not valid, and we are not sure about the genetic identity of *L. majoris.*

*Leucocytozoon* parasites from the wood warbler and the common chiffchaff were morphologically similar to *L. fringillinarum* (Figure 2u–ad). However, the phylogenetic analysis (Figure 3) showed that the *Leucocytozoon* lineages found in both hosts (WW6 and AFR205) do not cluster with the other lineages that have been formerly identified as *L. fringillinarum*, ZOLEU02 and TFUS04. On the other hand, these two lineages cluster together with *L. polynuclearis* (COLAUR01 and DRYALB01) (Figure 3, clade C), one of the parasite species that have been reported to develop in thrombocytes in North American woodpeckers (Piciformes) [2]. It is necessary to mention that *Leucocytozoon macleani* (possible synonym is *Leucocytozoon sabrazesi*) lineage GALLUS08, the parasite of chickens that inhabits thrombocytes, was placed in a different branch in the phylogenetic tree (Figure 3). However, *L. macleani* gametocytes develop in fusiform host cells, which is an important taxonomic character for *Leucocytozoon* parasite identification [5]. It is possible to say that the clade where our samples were placed represents parasites that deform the host cell into a roundish host-parasite complex and inhabit thrombocytes. Our phylogenetic analysis showed that the origin of cells inhabited by *Leucocytozoon* parasites likely is evolutionary informative and might be used in species taxonomy (Figure 3). However, it is too early to draw any conclusions. The lack of information is mainly because this issue was not the aim of previous studies with *Leucocytozoon* parasites. In addition, the low parasitemia frequently seen in wild birds together with the fact that merozoites and young gametocytes are not always present in the blood films of naturally infected birds, represents a big challenge for parasitologists. Investigations addressing this aspect of *Leucocytozoon* parasites biology should be encouraged in future research.

Noteworthy some of the parasite sequences found in the present study were genetically closely related to *L. fringillinarum* ZOLEU02 and TFUS04, even though morphologically they were different. *Leucocytozoon fringillinarum* was originally described in the common chaffinch *Fringilla coellebs* in England [5]; however, these two lineages were obtained from different hosts (*Zonotrichia leucophrys* and *Turdus fuscater*, respectively) and different regions (The United States of America and Colombia) [32,49]. These parasite lineages were linked to morphospecies based on the morphological features of gametocytes in blood films, but these two lineages are not closely related and might not represent the original *L. fringillarum* description. In other words, these identifications are likely wrong and should be revised in the future.

It is also important to remember that phylogenies are hypotheses, and frequently relatively small DNA fragments are used in phylogenetic analysis, which might not represent the true evolutionary history of investigated organisms [30,33,47]. This highlights the importance not only of including additional genes in the phylogenetic analysis but also of combining morphological features of other life cycle stages and development characteristics of *Leucocytozoon* parasites in the analysis.

Knowledge about host cells is an important aspect of parasite biology because it helps to understand or to address some of the effects of parasitic infections on their hosts. For example, avian *Plasmodium* parasites are well known for causing anemia, characterized by low haematocrit values, that is frequently found in infected individuals, especially during the acute stage of the disease [50,51]. Similar to what occurs with the erythrocytes and *Plasmodium* infection, if other cells are infected by parasites, their function might also be compromised. In other words, when thrombocytes are infected, their ability to form clots might be reduced, which can lead to excessive bleeding if the bird is injured. Similarly, if leukocytes are infected, the host immune system might face some difficulties identifying a pathogen and mounting an immune response against it. However, the host inflammatory response to parasitic infections is much more complex than that and assumptions should be carefully developed [52,53,54].

Even though understanding the burden of infection in vertebrate hosts is an important question to be answered, this problem has been poorly addressed for *Leucocytozoon* parasites. Additionally, it is expected that different parasite lineages will affect their avian hosts in different ways. In other words, some lineages might be more pathogenic than others. Yet, this is also avian host species dependent, since some lineages do not seem to be pathogenic for some bird species, while they can cause disease in other ones. This is known for other haemosporidian parasites. For example, *Plasmodium elongatum* (pERIRUB1) caused high mortality in experimentally infected canaries *Serinus canaria* [55]; *H. minutus* (hTURDUS2), a common parasite of blackbirds *T. merula*, is highly virulent in psittacine birds [56,57].

Reports about *Leucocytozoon* species pathogenicity are scarce for wild birds. At the tissue stage level, it was shown that *Leucocytozoon* sp. TURMER01 induce inflammatory reaction and necroses in infected organs [10]. Blood sample analysis shows that *Leucocytozoon* infections (unknown lineage) in barn owls (*Tyto alba*) can increase the total leukocyte count and the heterophil:lymphocyte ratio (H:L) [58], which is a known indicator of immune response to stress and illness in birds [59], as well as low haematocrit [58]. Contrary to that, *Leucocytozoon* infection (unknown lineage) in the American crows (*Corvus brachyrhynchos*) showed a slight increase in haematocrit levels, but this was not statistically significant [60]. In another case, *Leucocytozoon* infection (unknown lineage) in mallard ducks (*Anas platyrhynchos*) was not associated with changes in haematocrit levels of infected birds [61]. However, knowing that not all *Leucocytozoon* lineages will infect erythrocytes, their pathogenicity might not be related to low haematocrit levels, but to other changes in the blood cell parameters, which might lead to biased conclusions, including hypotheses that certain parasites can be even benign [61].

These findings highlight the importance of investigating other blood parameters when evaluating parasite pathogenicity, such as a total white blood cell count and the proportion of different leukocytes. This is particularly true because even the most often used haematological parameters, such as the H:L ratio, can be difficult to interpret and lead to wrong conclusions [59,62]. For example, cold stress can increase the H:L ratio, however, it comes back to normal values after several hours, or even severe life-threatening situations can reduce the number of heterophils, but it increases in a few hours when the situation returns to normal [59,62]. Microscopic analysis of blood films represents only one time point in the very dynamic response of the host to its environment. Thus, the combination of different parameters would provide more information on how the parasites can affect their host.

## 5. Conclusions

This study focused on the identification of host cells inhabited by certain *Leucocytozoon* parasite lineages during natural infections in wildlife and investigated if this character is phylogenetically important. We added new information to this subject and showed that *Leucocytozoon* parasites can inhabit different blood cells, such as erythrocytes, lymphocytes, and thrombocytes. The reason for this preference remains unclear; however, this biological feature seems to be phylogenetically important, and more attention should be given to it in future studies. Additionally, phylogenetic analysis using partial *cytb* gene sequences might be useful for the prediction of host cells that could be inhabited by certain parasite lineages.

## Figures and Tables

**Table 1:** Studied birds, *Leucocytozoon* lineages, and host cells where early blood stages were found.

Bird Species (Common Name)	*Leucocytozoon cytb* Lineage	Avian Host Cell	Co-infections with Other Haemosporidian Parasites (*cytb* Lineage)
*T. philomelos* (Song thrush)	STUR1	erythrocytes	*Plasmodium circumflexum* (TURDUS1)
*T. merula* (Blackbird)	NEVE01 + TURMER15	erythrocytes	*Plasmodium matutinum* (LINN1) *Haemoproteus minutus* (TURDUS2)
*S. borin* (Garden warbler)	- ^a^	erythrocytes	-
*C. caeruleus* (Blue tit)	PARUS4	lymphocytes	*P. circumflexum* (TURDUS1)
*P. sibilatrix* (Wood warbler)	WW6	thrombocytes	*Haemoproteus homopalloris* (PHSIB2)
*P. collybita* (Common chiffchaff)	AFR205	thrombocytes	*Haemoproteus asymmetricus* (TUPHI01)

^a^ Blood sample was not available for molecular analysis and parasite lineage identification.

## Data Availability

All generated sequence data were deposited in the NCBI GenBank and the MalAvi database. The parasite voucher preparations are available at Nature Research Centre, Vilnius, Lithuania, upon request.

## Supplementary Materials

The following supporting information can be downloaded at: https://www.mdpi.com/article/10.3390/pathogens12050712/s1. Supplementary Table S1: GenBank accession numbers of parasites cytochrome *b* gene sequences used in the Bayesian inference phylogenetic analysis. Supplementary Figure S1. Different types of blood cells in studied birds.
