# Supplementary material for: Host Cells of Leucocytozoon (Haemosporida, Leucocytozoidae) Gametocytes, with Remarks on the Phylogenetic Importance of This Character
Source: Pathogens. 2023 May 13;12(5):712. doi: 10.3390/pathogens12050712 (PMC10224414; doi:10.3390/pathogens12050712)
Supplement: Supplementary file 1 [file pathogens-12-00712-s001.zip › Supplementary figure S1.pdf]

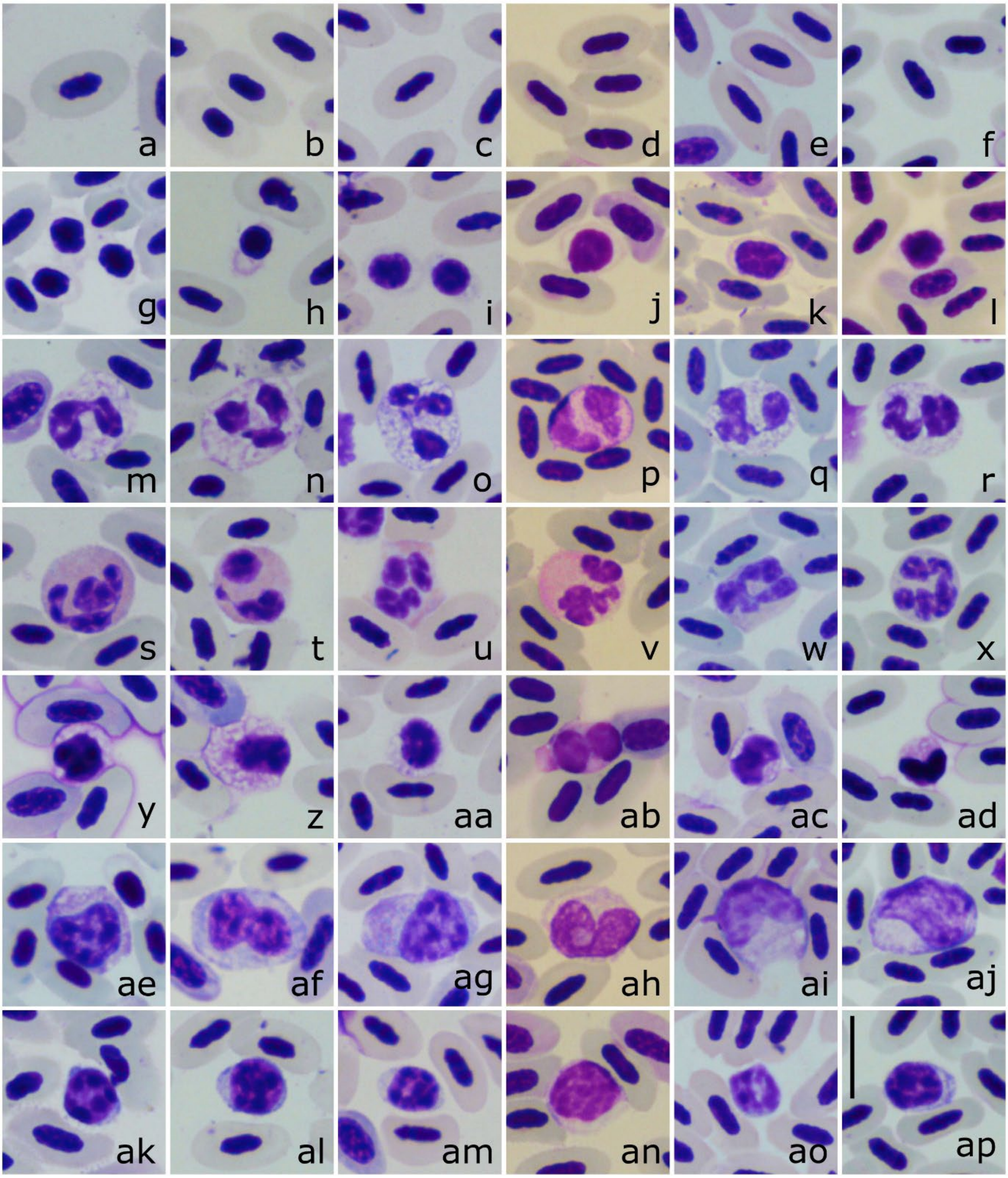

Supplementary Figure S1. Different types of blood cells in studied birds. Erythrocytes (**a-f**), thrombocytes (**g-l**), heterophils (**m-r**), eosinophils (**s-x**), basophils (**y-ad**), monocytes (**ae-aj**) and lymphocytes (**ak-ap**). Blood cells from song thrush *Turdus philomelos* (**a, g, m, s, y, ae, ak**), blackbird *Turdus merula* (**b, h, n, t, z, af, al**), garden warbler *Sylvia borin* (**c, i, o, u, aa, ag, am**), blue tit *Cyanistes caeruleus* (**d, j, p, v, ab, ah, an**), wood warbler *Phylloscopus sibilatrix* (**e, k, q, w, ac, ai, ao**), and common chiffchaff *Phylloscopus collybita* (**f, l, r, x, ad, aj, ap**). Methanol-fixed and Giemsa-stained blood films. Scale bar = 10 μm
